# Research on Motion Transfer Method from Human Arm to Bionic Robot Arm Based on PSO-RF Algorithm

**DOI:** 10.3390/biomimetics10060392

**Published:** 2025-06-11

**Authors:** Yuanyuan Zheng, Hanqi Zhang, Gang Zheng, Yuanjian Hong, Zhonghua Wei, Peng Sun

**Affiliations:** 1 School of Mechanical and Energy Engineering, Zhejiang University of Science and Technology, Hangzhou 310023, China; zhengyy@zust.edu.cn (Y.Z.);; 2 College of Mechanical Engineering, Zhejiang University of Technology, Hangzhou 310023, China; 3 College of Mechanical and Electrical Engineering, China Jiliang University, Hangzhou 310018, China; 4 Zhejiang LINIX Motor Co., Ltd., Dongyang 322118, China

**Keywords:** bionic robot arm, motion transfer method, joint space mapping, PSO-RF algorithm

## Abstract

Although existing motion transfer methods for bionic robot arms are based on kinematic equivalence or simplified dynamic models, they frequently fail to tackle dynamic compliance and real-time adaptability in complex human-like motions. To address this shortcoming, this study presents a motion transfer method from the human arm to a bionic robot arm based on the hybrid PSO-RF (Particle Swarm Optimization-Random Forest) algorithm to improve joint space mapping accuracy and dynamic compliance. Initially, a high-precision optical motion capture (Mocap) system was utilized to record human arm trajectories, and Kalman filtering and a Rauch–Tung–Striebel (RTS) smoother were applied to reduce noise and phase lag. Subsequently, the joint angles of the human arm were computed through geometric vector analysis. Although geometric vector analysis offers an initial estimation of joint angles, its deterministic framework is subject to error accumulation caused by the occlusion of reflective markers and kinematic singularities. To surmount this limitation, this study designed five action sequences for the establishment of the training database for the PSO-RF model to predict joint angles when performing different actions. Ultimately, an experimental platform was built to validate the motion transfer method, and the experimental verification showed that the system attained high prediction accuracy (R^2^ = 0.932 for the elbow joint angle) and real-time performance with a latency of 0.1097 s. This paper promotes compliant human–robot interaction by dealing with joint-level dynamic transfer challenges, presenting a framework for applications in intelligent manufacturing and rehabilitation robotics.

## 1. Introduction

As technology progresses, bionic robots have rapidly developed, demonstrating great potential in fields such as intelligent manufacturing, field operation, and medical rehabilitation [1,2,3]. The human arm has a complex motion, containing multi-joint forward flexion and backward extension, abduction and adduction, and other motions [4]. Therefore, when the bionic robotic arm performs tasks it needs to maintain similar dynamic behaviors to the human body motion, and the traditional robotic arm trajectory planning methods are difficult to adapt to highly dynamic and complex motions [5]. As such, it is necessary to analyze the human motion, extract the motion characteristics of the human arm during different motions, and apply them in the trajectory planning and control algorithm of a bionic robot arm.

Motion capture (Mocap) systems are employed to acquire kinematic trajectories of human body motions to analyze or reproduce complex motion trajectories [6,7]. Human Mocap can be categorized into the following four specialized systems tailored to distinct application scenarios: inertial Mocap systems [8], ultrasonic Mocap systems [9], vision-based Mocap systems [10], and optical Mocap systems [11,12].

Based on the Mocap systems, Jia et al. [13] developed a robotic arm trajectory planning method leveraging the learning and generalization capabilities of Dynamic Movement Primitives (DMPs), demonstrating 54.2% decrease in average trajectory deviation. Yu et al. [14] proposed a robotic skill-learning framework integrating motion and impedance features, utilizing electromyography (EMG) to estimate human upper-limb stiffness and employing DMPs for the dual-modal encoding of kinematic trajectories and impedance parameters. This method was validated in a KINOVA robotic water-pumping task, achieving a 96.2% success rate, 37.5% reduction in peak human–robot interaction forces, and limiting trajectory tracking errors to within ±1.8 mm. Vuga et al. [15] presented a real-time humanoid motion transfer system using low-cost RGB-D sensing, enabling coordinated improvement of motion imitation fidelity.

Existing studies predominantly focus on end-effector trajectory transfer while neglecting the dynamic transfer of joint angles. The continuity of human joint motion is crucial for achieving compliant and coordinated arm movements. To enhance the compliance of arm motion transfer, Zhao et al. [16] developed a human-like motion planner leveraging Human Arm Motion Patterns (HAMPs) for robotic handover tasks, implementing task decomposition, HAMP feature extraction, and duration-optimized primitive sequencing. Experimental validation on the KUKA IIWA robot demonstrates that the generated trajectories accurately transfer the joint coordination characteristics inherent in human arm movements. Current methodologies predominantly rely on manual parameter tuning, exhibit limited generalization capability, and suffer from a lack of interpretability in deep learning models, thereby compromising the robustness of bionic robotic arms in complex operational scenarios.

Diverging from prior research on motion transfer methods for bionic robot arms, this paper collects human arm motion data through a high-precision optical Mocap system, extracts joint angle features by the geometric vector method, uses modified particle swarm optimization to optimize the hyperparameters of the random forest model, establishes a mapping model of human arm motion–joint angle, and verifies the motion transfer method through prototype experiment.

The structure of this paper is organized as follows: Section 1 delineates fundamental challenges in human-to-robot motion transfer through a comprehensive literature review, establishing theoretical foundations for bionic robot arm control. Section 2 arranges the motion capture system and incorporates Kalman filtering and an RTS smoother for trajectory processing. Section 3 details the geometric vector analysis framework for human arm kinematics modeling, and introduces the hybrid PSO-RF algorithm architecture, specifying the particle swarm optimization parameters and random forest configuration strategies. Section 4 constructs the experimental platform and verifies the proposed PSO-RF algorithm on the prototype of the bionic robot arm. The superiority of the algorithm is verified through a comparison with existing methods. Section 5 critically discusses the advantages and disadvantages of the research work. Section 6 describes the conclusion of the article.

## 2. Motion Capture Solution

### 2.1. Arrangement of the Mocap System

**Figure 1:**
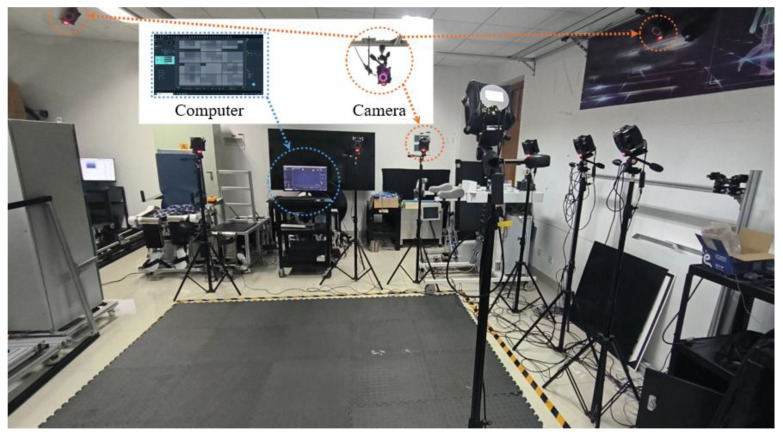
Global view of the Mocap system.

To achieve the real-time capture of complex human arm motions, this paper employs the Nokov Mocap System [17], configured with twelve infrared Mars-12H cameras (100 Hz frame rate, ±0.08 mm accuracy), a host computer, and some reflective markers, as shown in Figure 1. The proprietary XINGYING 3.2 software suite provides 3D coordinate trajectory data of the reflective markers, and the end-to-end processing latency can be controlled within 3 ms.

### 2.2. Motion Capture Recognition

**Figure 2:**
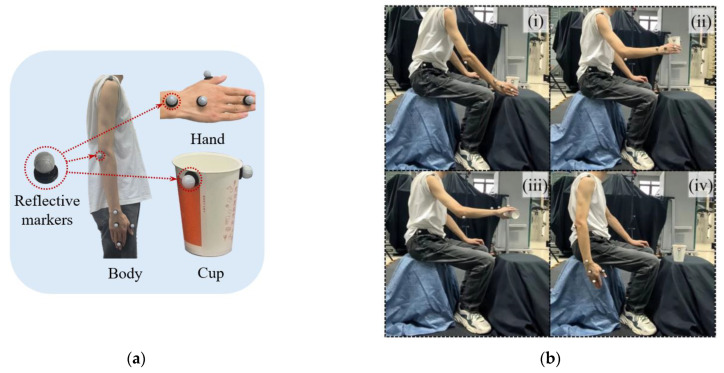
Capture points and capture actions: (**a**) reflective markers; (**b**) captured actions.

The optical Mocap system relies on reflective markers to capture the motion of objects. By reasonably arranging the reflective markers at the positions of the human arm, the rotation angle of the human arm joints can be calculated according to the geometric vector method. Reflective makers are arranged at the hip, shoulder, elbow, wrist joint, and palm, respectively. The hip joint reflective maker serves as the coordinate origin of the human arm motion model. The arrangement position of reflective markers as shown in Figure 2a.

Under proper lighting conditions, with potential hazards eliminated and the safety of the experimental environment ensured, the motion capture experiment was set up to simulate the human action of pouring water. The single-group motion flow is illustrated in Figure 2b, and the specific experimental steps are as follows:

(i) Vertically place the hand beside the cup.
(ii) Extend the thumb, grip the cup, and lift it to a height of one palm.
(iii) Tilt the wrist 90 degrees and maintain this position for 2 s, then rotate the wrist 90 degrees in the opposite direction to return it to the neutral position and lower the hand.
(iv) Straighten the palm and return to the initial position.

When repeatedly collecting the same motions, the participant should keep the same state to enhance the data stability.

### 2.3. Coordinate Trajectory Processing

The acquired trajectories of the reflective makers during human arm motion exhibit high-frequency noise due to intrinsic physiological tremors and extrinsic vibrations caused by environmental interactions. Because the noise exhibits Gaussian characteristics, and the trajectories adhere to linear kinematic constraints, it can be seen as a linear Gaussian (LG) system. The discrete-time state-space model [18] can be described as follows:

(1) $$\left(\begin{array}{l} x_{k}=F_{k|k-1}x_{k-1}+w_{k},\;w_{k}\sim N(0,Q_{k})\\ z_{k}=H_{k}x_{k}+v_{k},\;v_{k}\sim N(0,R_{k}) \end{array}\right.$$

where $x_{k}$ is the state vector, $z_{k}$ is the trajectory of markers, $F_{k|k-1}$ is the state transition matrix, $H_{k}$ is the observation matrix, $Q_{k}$ is the process noise covariance, $R_{k}$ is the measurement noise covariance, $w_{k}$ is the process noise, and $v_{k}$ is the measurement noise.

According to Kalman filtering [19], the iteration of the model from Equation (1) can be described as follows:

(i) Prediction Step:

(2) $${\hat{x}}_{k|k-1}=F_{k}{\hat{x}}_{k-1|k-1}\;P_{k|k-1}=F_{k}P_{k-1|k-1}F_{k}^{⊤}+Q_{k}$$

(ii) Prediction Step:

(3) $$K_{k}=P_{k|k-1}H_{k}^{⊤}{\left(H_{k}P_{k|k-1}H_{k}^{⊤}+R_{k}\right)}^{-1}\;{\hat{x}}_{k|k}={\hat{x}}_{k|k-1}+K_{k}(z_{k}-H_{k}{\hat{x}}_{k|k-1})\;P_{k|k}=(I-K_{k}H_{k})P_{k|k-1}$$

**Figure 3:**
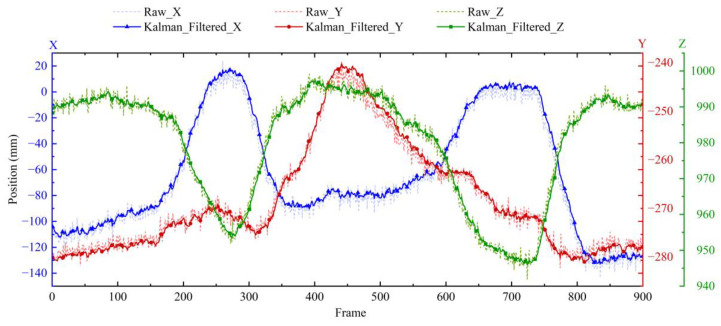
Before and after x-y-z position filtering of shoulder joints with Kalman filter.

The shoulder joint coordinates obtained in the experiment of Figure 2b are processed by Kalman filtering, and the obtained comparison image is shown in Figure 3.

The sampling frequency of the Mokov Mocap system is 100 Hz, meaning that every 100 frames represent 1 s. After filtering the raw data, the estimated result is still different from the expected result, and the Kalman filtered trajectories have a slight phase lag. A Rauch–Tung–Striebel (RTS) smoother [20] was employed to further reduce the fluctuation of the estimated result and maintain the consistency of the phase.

Define $C_{k}$ as the smooth gain matrix, and as such the smooth gain can be calculated as follows:

(4) $$C_{k}=P_{k|k}F_{k+1}^{⊤}{\left(P_{k+1|k}\right)}^{-1}$$

Update the smooth state as follows:

(5) $${\hat{x}}_{k|N}={\hat{x}}_{k|k}+C_{k}({\hat{x}}_{k+1|N}-{\hat{x}}_{k+1|k})\;\;\;\;\;\;\;P_{k|N}=P_{k|k}+C_{k}(P_{k+1|N}-P_{k+1|k})C_{k}^{⊤}$$

where N is the total number of trajectory sampling points.

**Figure 4:**
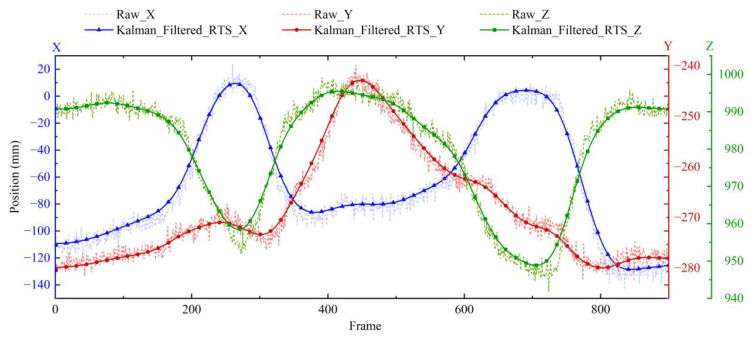
Before and after x-y-z position filtering of shoulder joints with RTS smoother.

After employing an RTS smoother, the obtained comparison image is shown in Figure 4.

According to Figure 4, the filtered data maintains good phase consistency compared to the original data and eliminates the original noise.

## 3. Methodology

### 3.1. Calculation of the Human Arm Model Joint Angles

Through the motion capture system, the 3D coordinates of reflective markers can be obtained when the subject performs different tasks. To achieve motion transfer between the arm and the robotic arm, it is necessary to acquire the joint angle data of the arm based on the information of the reflective markers.

**Figure 5:**
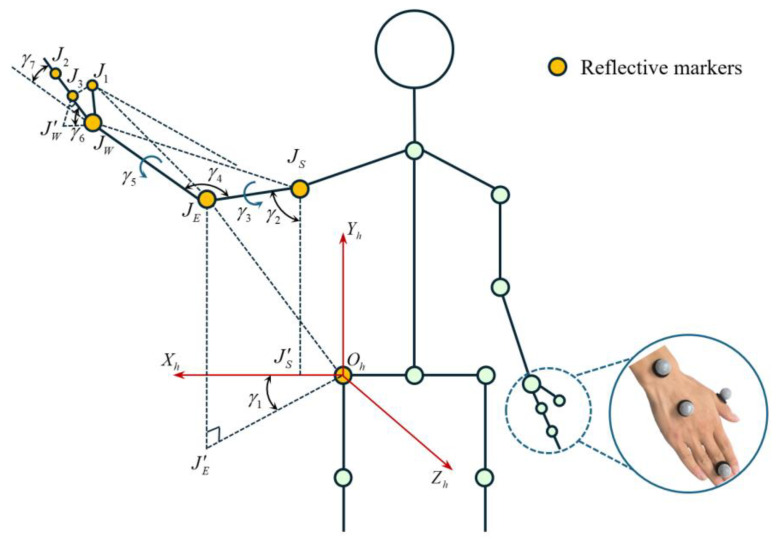
Schematic diagram of the joint angles of the human arm model.

The establishment of the arm model is shown in Figure 5. The coordinate system $O_{h}-X_{h}Y_{h}Z_{h}$ is established by taking the right hip joint $O_{h}$ as the coordinate origin. Where $J_{S}$ is the shoulder joint, $J_{E}$ is the elbow joint, $J_{W}$ is the wrist joint, and $J_{1}$, $J_{2}$, and $J_{3}$ are the reflective markers at the palm, respectively.

$\gamma_{1}$ is the angle between plane $X_{h}O_{h}Y_{h}$ and plane $J_{E}O_{h}{J^{\prime }}_{E}$, representing the flexion and extension of the shoulder joint, which can be expressed as follows:

(6) $$\gamma_{1}=\langle \vec{OX_{h}}\times \vec{OY_{h}},\vec{OJ_{E}}\times \vec{O{J^{\prime }}_{E}}\rangle$$

where point ${J^{\prime }}_{E}$ is the mapping point plane $X_{h}O_{h}Z_{h}$, and so construct a line through point $J_{S}$, parallel to line $O_{h}X_{h}$, intersecting plane $X_{h}O_{h}Z_{h}$ at point ${J^{\prime }}_{S}$.

$\gamma_{2}$ is the angle between line $J_{S}J_{E}$ and line $J_{S}{J^{\prime }}_{S}$, representing the adduction and abduction of the shoulder joint, and this can be expressed as follows:

(7) $$\gamma_{2}=\langle \vec{J_{S}J_{E}},\vec{J_{S}{J^{\prime }}_{S}}\rangle$$

$\gamma_{3}$ is the angle between plane $J_{S}J_{E}J_{W}$ and plane $X_{h}O_{h}Y_{h}$, representing the internal and external rotation of the shoulder joint, which can be expressed as follows:

(8) $$\gamma_{3}=\langle \vec{J_{S}J_{E}}\times \vec{J_{E}J_{W}},\vec{OZ_{h}}\rangle$$

$\gamma_{4}$ is the angle between line $J_{S}J_{E}$ and line $J_{E}J_{W}$, representing the flexion and extension of the elbow joint, which can be expressed as follows:

(9) $$\gamma_{4}=\langle \vec{J_{S}J_{E}},\vec{J_{E}J_{W}}\rangle$$

$\gamma_{5}$ is the angle between plane $J_{E}J_{W}J_{1}$ and plane $X_{h}O_{h}Y_{h}$, representing the pronation and supination of the forearm, which can be expressed as follows:

(10) $$\gamma_{5}=\langle \vec{J_{E}J_{W}}\times \vec{J_{W}J_{1}},\vec{OZ_{h}}\rangle$$

$\gamma_{6}$ is the angle between plane $J_{W}J_{1}J_{3}$ and line $J_{E}J_{W}$, representing the dorsiflexion and palmar flexion of the wrist joint, which can be expressed as follows:

(11) $$\gamma_{6}=\langle \vec{J_{W}J_{1}}\times \vec{J_{W}J_{3}},\vec{J_{E}J_{W}}\rangle$$

$\gamma_{7}$ is the angle between line $J_{W}J_{2}$ and line $J_{E}J_{W}$, representing the radial and ulnar deviation of the wrist joint, which can be expressed as follows:

(12) $$\gamma_{7}=\langle \vec{J_{W}J_{2}},\vec{J_{E}J_{W}}\rangle$$

The rotation angles of the seven joints of the human arm can be calculated by Equations (6)–(12). To obtain accurate calculation results, the participant needs to avoid sudden changes during arm motion to prevent data loss.

While geometric vector analysis offers a deterministic framework for calculating joint angles based on spatial coordinates, its reliance on idealized assumptions limits its applicability in real-world scenarios. Specifically, incomplete sensor data, noise interference, and singular configurations in complex human motions, such as extreme joint rotations or overlapping limb trajectories, can degrade the accuracy of purely geometric solutions [21,22]; hence, this paper employs a joint angle prediction model to address these challenges.

### 3.2. PSO-RF Algorithm

To predict the joint angles of the human arm in different motions, this paper sets up five different sets of motions and analyzes the rotation of the shoulder, elbow, and wrist joint of a human arm, as shown in Table 1.

Before the formal start of the experiment, the participant should be acquainted with the entire experimental procedure and safety precautions. The participant should execute the experimental motions in the Mokov Mocap system, and complete each preset within 10 s.

**Figure 6:**
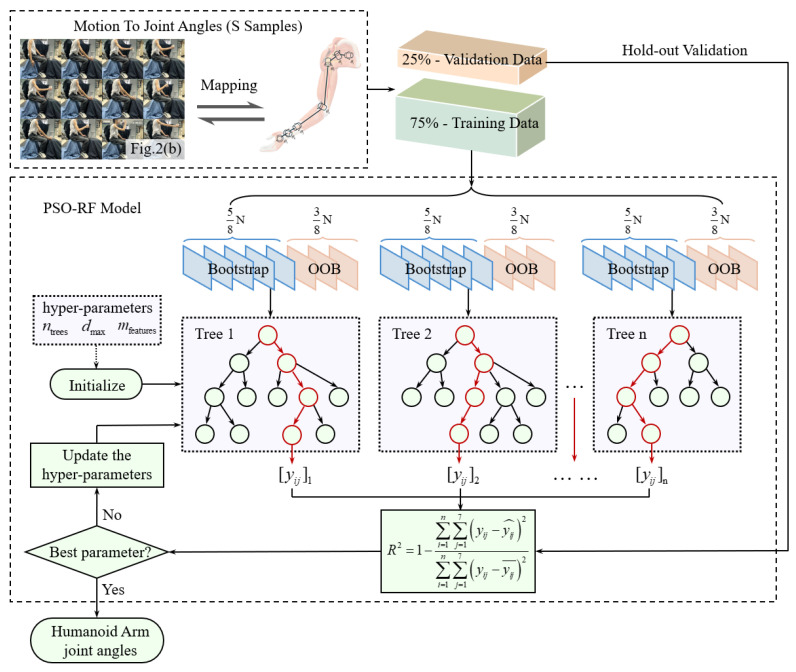
PSO-RF structure of joint angles prediction model.

The focus of the joint angle prediction model is to establish a regression model that takes inputs from human arm trajectories to predict the various joint angles when performing complex motions. While random forest (RF) demonstrates inherent advantages in handling nonlinear biomechanical relationships through ensemble learning, its manual hyperparameter tuning proves inadequate due to its inherent constraints, which are its subjective dependency on empirical settings and its suboptimal generalization performance under motion variability. To address these limitations, this study develops a hybrid PSO-RF architecture [23,24] that leverages the global search capability of particle swarm optimization (PSO) to optimize the hyperparameters of the RF algorithm. The PSO-RF training architecture is shown in Figure 6.

Database establishment:

Kinematic data were collected using the Mokov Mocap system under controlled laboratory conditions. Reflective markers were pasted onto the experimental participants in the configuration shown in Figure 5 to track the 3D trajectories during the predefined actions listed in Table 1. The participant was required to perform standardized motions, and each action in the five action sequences in Table 1 was repeated 50 times, with each single action to be completed within 10 s. In total, 250 sets of training data were obtained, with 75% used for training and 25% for prediction.

Initial:

Define $n_{\mathrm{trees}}$, $d_{\max}$, and $m_{\mathrm{features}}$ as the number, maximum degree, and characteristic sampling ratio of the RF model, respectively, $X_{i}\in {\mathbb{R}}^{7\times 3}={\left[O_{h},J_{S},J_{E},J_{W},J_{1},J_{2},J_{3}\right]}^{T}$ as the input of the PSO-RF model, and $\Theta_{i}\in {\mathbb{R}}^{7}={\left[\gamma_{1},\gamma_{2},\gamma_{3},\gamma_{4},\gamma_{5},\gamma_{6},\gamma_{7}\right]}^{T}$ as the output of the PSO-RF model. Therefore, the training dataset can be described as $S=\left\{\left(X_{i},\Theta_{i}\right)\right\}$.

The hyperparameters of the RF model are defined as the following initial particle positions:

(13) $$\vartheta_{\mathrm{PSO}}={\left[n_{\mathrm{trees}},d_{\max},m_{\mathrm{features}}\right]}^{T}$$

According to the input and output characteristics, the constraint range is specified as follows:

(14) $$n_{\mathrm{trees}}\in [50,200],d_{\max}\in [5,30],m_{\mathrm{features}}\in [0.2,0.8]$$

**Validation:**

To evaluate the generalization performance of the PSO-RF model, the dataset is partitioned using hold-out validation [25]. Specifically, 75% of the experimental data (denoted as $S_{train}=\left\{\left(X_{i}^{train},\Theta_{i}^{train}\right)\right\}$) is randomly selected for model training, while the remaining 25% (denoted as $S_{valid}=\left\{\left(X_{i}^{valid},\Theta_{i}^{valid}\right)\right\}$) is reserved for validation.

Given the inherent complexity of human arm motion transfer, it is critical to prioritize explained variance and generalization in the trajectory mapping framework. To address these requirements, R squared (R^2^) is adopted as the optimization objective, and the fitness function is constructed as follows:

(15) $$R^{2}=1-\frac{\sum_{i=1}^{n}{\left(\gamma_{i}-{\hat{\gamma }}_{i}\right)}^{2}}{\sum_{i=1}^{n}{\left(\gamma_{i}-\bar{\gamma }\right)}^{2}}\;F(\vartheta_{\mathrm{PSO}})=R^{2}(\Theta_{i},{\hat{\Theta }}_{i})$$

where $\gamma_{i}$ is the measured angles, ${\hat{\gamma }}_{i}$ is the predicted angles, and $\bar{\gamma }$ is the average value of the measured angles.

**Update:**

For the particle i at the iteration t, the following can be obtained:

(16) $$v_{i}(t+1)=\omega v_{i}(t)+c_{1}r_{1}\left(p_{i}^{\mathrm{best}}-\vartheta_{i}^{\left(t\right)}\right)+c_{2}r_{2}\left(g^{\mathrm{best}}-\vartheta_{i}^{\left(t\right)}\right)\;\vartheta_{i}(t+1)=\vartheta_{i}(t)+v_{i}(t+1)$$

where ω is the inertia weight; $v_{i}$ is the velocity of particle; $c_{1}$ is the cognitive acceleration coefficient; $c_{2}$ is the social acceleration coefficient; $r_{1}$, $r_{2}$ are the random exploration factors, such that there is $r_{1},r_{2}\in [0,1]$; $p_{i}^{\mathrm{best}}$ is the personal best position; and $g^{\mathrm{best}}$ is the global best position.

In this system, the convergence threshold e was set to 0.005, the maximum iteration $T_{\max}$ was set to 150 with acceleration coefficients $c_{1}$ = $c_{2}$ = 2.05, and the inertia weight ω was set to 0.7.

After the above analysis, the pseudocode of the PSO-RF algorithm in this paper is shown in Algorithm 1.

**Algorithm 1.** The pseudocode of the PSO-RF algorithm.Pseudocode: PSO-RF algorithm for joint angles prediction (python)# Particle initialization        NP = 50, t_max = 150, e = 0.005, w = 0.7, c1 = c2 = 2.05  # PSO        rf_para = {‘n_trees’:[50, 200], ‘d_max’:[5, 30], ‘m_features’:[0.2, 0.8]}  # RF# Swarm optimization        particles = [{‘pos’:[randint(n_trees), randint(d_max), random(m_features)],                               ‘vel’:[0, 0, 0], ‘pbest’:[], ‘pfit’: −inf})        gbest = {‘pos’:[], ‘fit’: −inf}, prev_fit = −inf        for i = 1 to t_max:            for p in particles:                rf = RandomForest (n_trees = p.pos [0], d_max = p.pos [1], m_features = p.pos [2])                X_train, X_val, y_train, y_val = split(S, 0.75)                rf.fit(X_train, y_train)  # Train RF model        y_pred = rf.predict(X_val)                R2 = 1 − (sum((y_val−y_pred)**2)/sum((y_val−mean(y_val))**2))  #R^2^ fitness                if R2 > p[‘pfit’]: p.update(pfit = R2, pbest = p[‘pos’])                if R2 > gbest[‘fit’]: gbest.update(fit = R2, pos = p[‘pos’])    # Update bests# Update particle dynamics        for p in particles:            for i = 0 to 2                r1, r2 = random(), random()                p[‘vel’][i] = w*p[‘vel’][i] + c1*r1*(p[‘pbest’][i]−p[‘pos’][i]) +                 c2*r2*(gbest[‘pos’][i] − p[‘pos’][i])                p[‘pos’][i] = clamp(p[‘pos’][i] + p[‘vel’][i], rf_para [i])            if abs(gbest[‘fit’] − prev_fit) < e: break            prev_fit = gbest[‘fit’]        return gbest[‘pos’]

### 3.3. Analysis of Joint Angles Prediction Results

**Figure 7:**
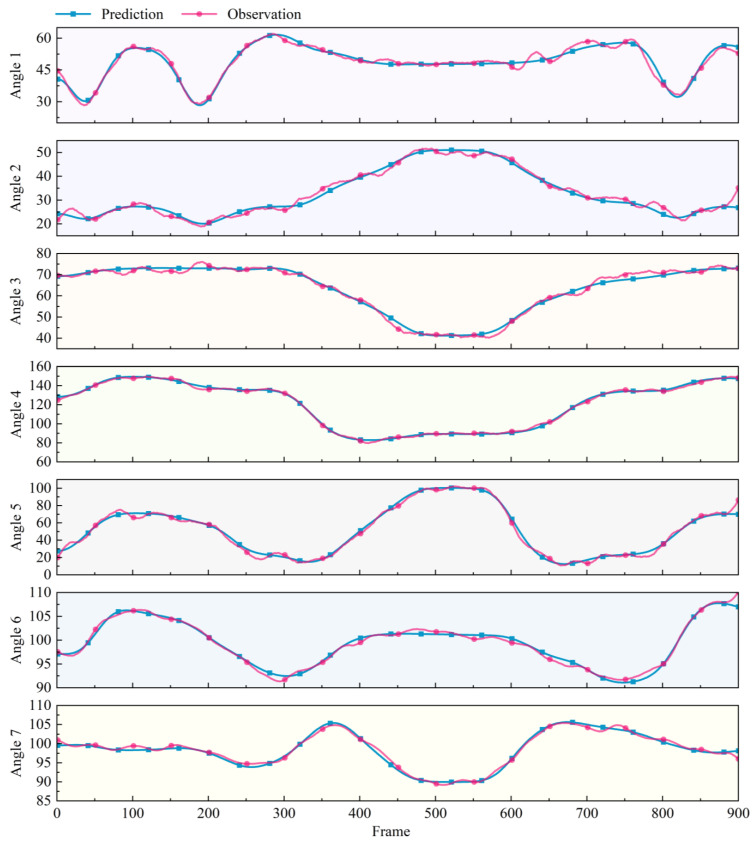
Comparison diagram of the predicted and actual observed angles.

The drink water action set in Table 1 was used as the test input for the trained PSO-RF model to predict joint angles, and the predicted angles and the actual observed angles are shown in Figure 7.

**Figure 8:**
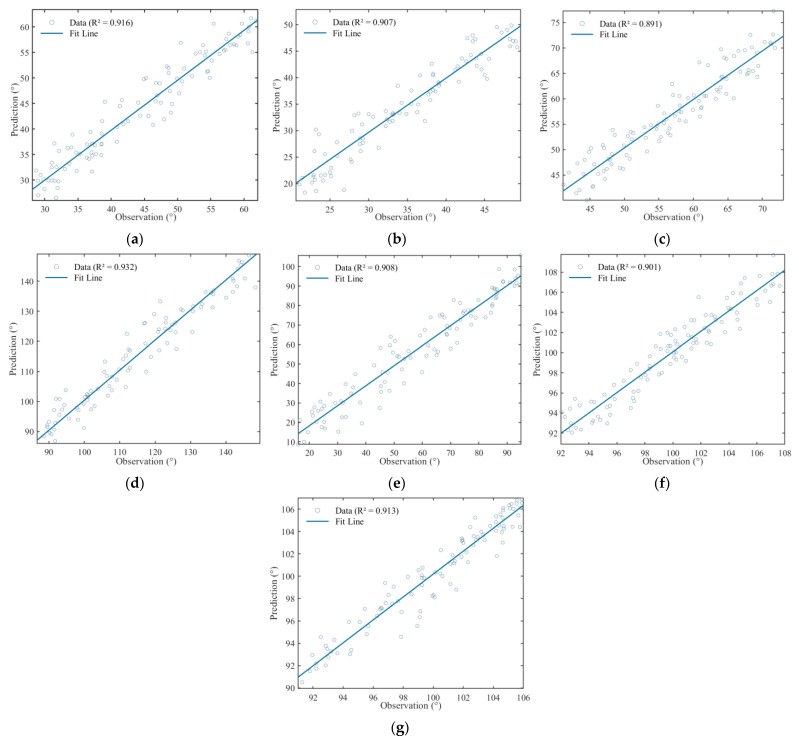
Prediction effect plots obtained for seven angles: (**a**) angle 1; (**b**) angle 2; (**c**) angle 3; (**d**) angle 4; (**e**) angle 5; (**f**) angle 6; and (**g**) angle 7.

The trends of the two curves in the figure are almost identical. Angle 4 represents the rotation angle of the elbow joint, whose movement is relatively simple and has the best prediction effect. The remaining angles also achieve good prediction results. The effect analysis of the seven prediction angles as shown in Figure 8.

## 4. Experiment

### 4.1. Joint Mapping Analysis

Human arm motions are completed by the shoulder, elbow, and wrist joints, which have different degrees of freedom and work together to operate complex motions. The shoulder joint has three degrees of freedom, the elbow joint has one degree of freedom, and the wrist joint has three degrees of freedom [26,27]. To realize the motion transfer between the human arm and the bionic robot arm, it is necessary to conduct a degree-of-freedom analysis of the bionic robot arm.

**Figure 9:**
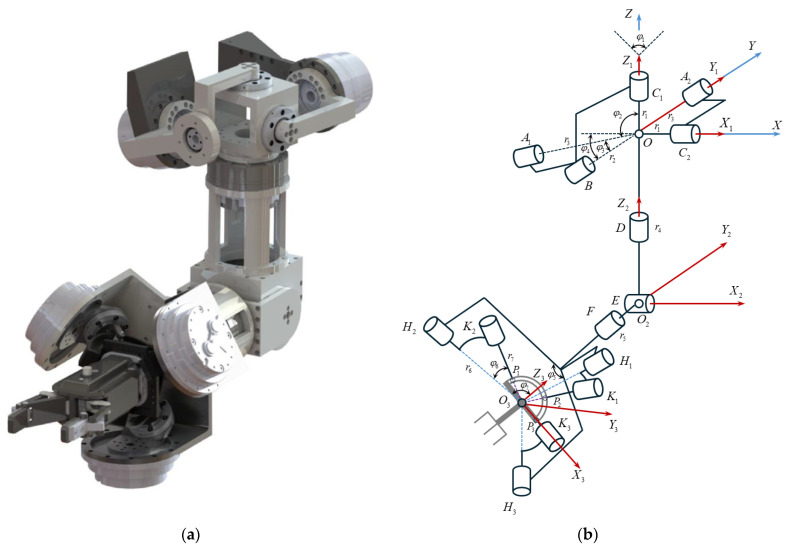
Bionic robot arm: (**a**) 3D model; (**b**) structural diagram [28].

The bionic robot arm used in the motion transfer experiment has been designed to mimic the shoulder, elbow, and wrist joints. The 3D model and structural diagram of the bionic robot arm are shown in Figure 9.

**Figure 10:**
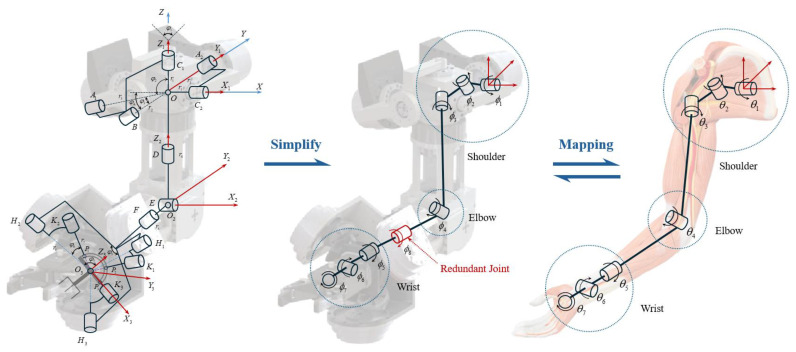
Joint mapping analysis of the bionic robot arm.

In previous work [28] we have solved the kinematics of the hybrid bionic robot arm and it can be simplified into a highly humanoid arm structure, as shown in Figure 10.

The bionic robot arm has a redundant degree of freedom (DOF) at the forearm, which is the same as the radial deviation/ulnar deviation at the wrist joint. After removing the redundant degree of freedom, its actual degrees of freedom are completely consistent with those of the human arm. Through kinematic and DOF analysis the motion transfer between the bionic robot arm and the human arm in the joint space can be realized.

### 4.2. Arm–Bionic Robot Arm Motion Tranfer Experiment

**Figure 11:**
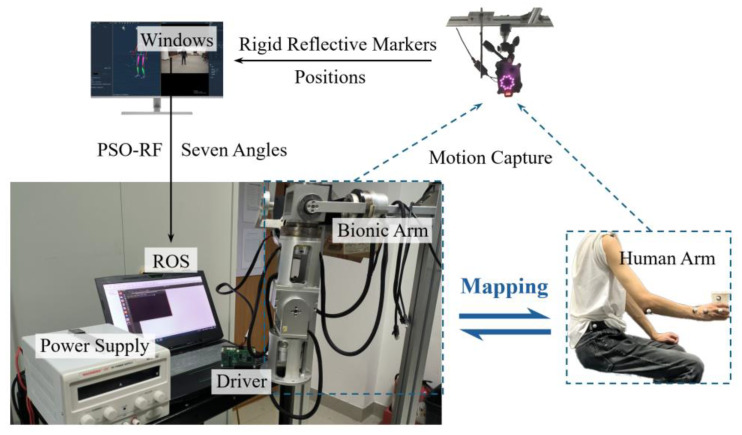
The experimental platform construction.

The experimental platform construction is shown in Figure 11. The participant pasted on the reflective markers in the form of Figure 2a and Figure 5, performing the motions set in Table 1. The Mokov Mocap system captured the trajectory signals and transmitted them to the host Windows computer software XINGYING 3.2. The filtered trajectory signals were processed through the PSO-RF model to generate predicted joint angle profiles. These profiles were converted into control commands of the robotic operation system (ROS), which orchestrated real-time actuation of the bionic robot arm, thus achieving the human arm motion transfer experiment.

**Figure 12:**
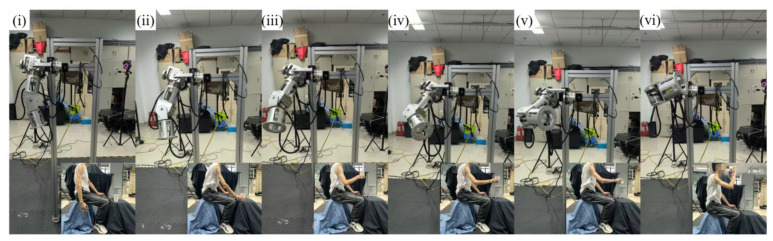
Experiment on motion transfer of bionic robot arm. (**i**) initial state; (**ii**) take the cup; (**iii**) lift the cup; (**iv**) flat the cup; (**v**) pour water; (**vi**) drink water.

To verify the effect of human arm motion transfer we randomly arranged and combined the sub-motions of the five motions in Table 1 to obtain the random experimental motion, as shown in Figure 12.

**Figure 13:**
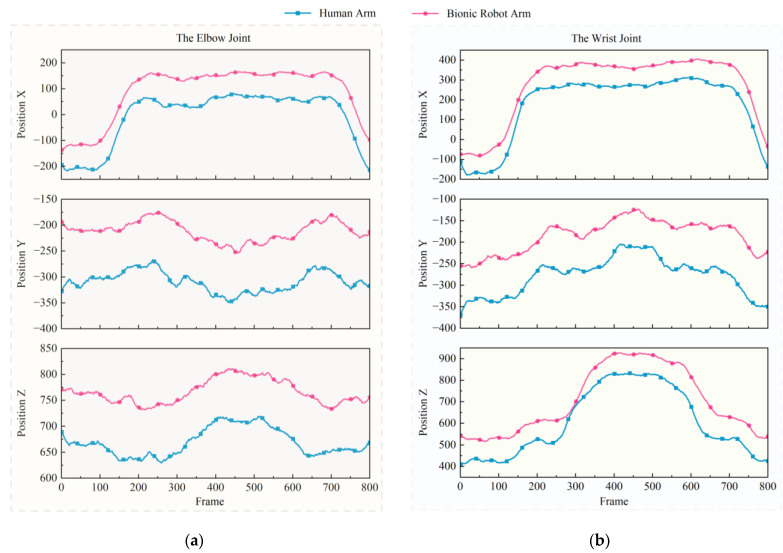
Trajectory similarity comparison: (**a**) the elbow joint; and (**b**) the wrist joint.

The sampling frequency of the Mokov Mocap system is 100 Hz, meaning that every 100 frames represent 1 s, and so during the 10 s motion execution the central 8 s interval (1–9 s) was analyzed to perform a comparative assessment of the 3D positional trajectories between human arm and the bionic robot arm, focusing on the elbow and wrist joints, as shown in Figure 13.

To quantitatively evaluate the temporal–spatial consistency between the human arm and the bionic robot arm trajectories, mean-centered dynamic time warping (mean-centered DTW) was employed as a similarity metric. Unlike traditional Euclidean distance measures, mean-centered DTW is robust to temporal misalignments and phase shifts by nonlinearly warping the time axis to minimize the cumulative distance between two sequences [29,30].

Define $X=\left\{x_{1},x_{2},\ldots ,x_{800}\right\}$ as the trajectory of the elbow joint of the human arm in the *z* direction and $Y=\left\{y_{1},y_{2},\ldots ,y_{800}\right\}$ as the trajectory of the elbow joint of the bionic robot arm in the *z* direction. Subsequently, these two trajectories are subtracted from their own mean values to eliminate the following overall offset:

(17) $$\left\{\begin{array}{c} {\tilde{x}}_{i}=x_{i}-\frac{1}{N}\sum_{i=1}^{N}x_{i}\\ {\tilde{y}}_{j}=y_{j}-\frac{1}{N}\sum_{j=1}^{N}y_{j} \end{array}\right.$$

The mean-centered DTW distance $d_{\text{Centered-DTW}}$ is computed as follows:

(18) $$d_{\text{Centered-DTW}}(X,Y)=\underset{W}{\min}\left(\sum_{\left(i,j\in W\right)}d({\tilde{x}}_{i},{\tilde{y}}_{j})\right)$$

where W represents the optimal warping path satisfying boundary, monotonicity, and continuity constraints, and $d({\tilde{x}}_{i},{\tilde{y}}_{j})$ is the Euclidean distance [31].

The system latency is computed by analyzing temporal offsets between corresponding peaks in the human arm trajectory $X=\left\{x_{1},x_{2},\ldots ,x_{800}\right\}$ and the bionic robot arm trajectory $Y=\left\{y_{1},y_{2},\ldots ,y_{800}\right\}$. Peak detection [32] was employed to identify the timestamps of all peaks in these trajectories, which is as follows:

(19) $$\mathrm{For}\;\mathrm{human}\;\mathrm{arm}\;\mathrm{peaks}\;\hat{X}=\left\{{\hat{x}}_{1},{\hat{x}}_{2},\ldots ,{\hat{x}}_{N}\right\}\;\mathrm{For}\;\mathrm{bionic}\;\mathrm{robot}\;\mathrm{arm}\;\mathrm{peaks}\;\hat{Y}=\left\{{\hat{y}}_{1},{\hat{y}}_{2},\ldots ,{\hat{y}}_{M}\right\}$$

If N≠M, truncate the longer sequence to align the datasets, ensuring one-to-one correspondence between human and bionic robot arm peaks. For each matched peak pair $\left({\hat{x}}_{i},{\hat{y}}_{i}\right)$, compute the following temporal difference $\Delta t_{i}$:

(20) $$\Delta t_{i}=t_{{\hat{x}}_{i}}-t_{{\hat{y}}_{i}}$$

Then calculate the mean latency across all matched peaks by performing the following:

(21) $$\Delta t_{avg}=\frac{1}{n}\sum_{i=1}^{n}\Delta t_{i}$$

where *n* is the number of matched peak pairs and $\Delta t_{avg}$ is the calculated system latency.

**Figure 14:**
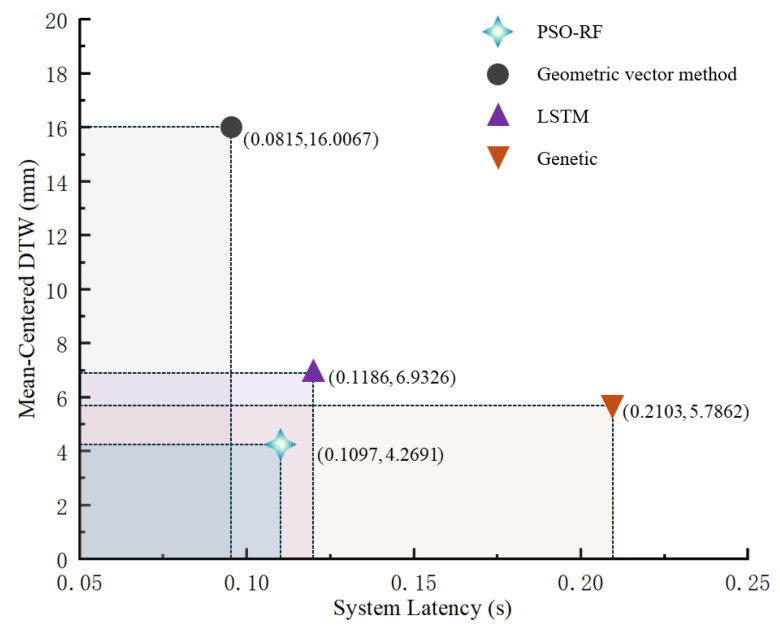
Mean-centered DTW and system latency comparison.

To verify the advances of the proposed PSO-RF algorithm, experiments were conducted using the PSO-RF algorithm, the long short-term memory (LSTM) algorithm [33], the genetic algorithm [34], and the geometric vector method, respectively. Figure 14 compared the mean-centered DTW values and system delays associated with the human arm trajectory in the *z*-direction of the elbow joint and the trajectory of the bionic robot arm in the *z*-direction among the above four methods.

As shown in Figure 13 and Figure 14, the proposed PSO-RF algorithm demonstrates high trajectory similarity between the human arm and the bionic robot arm. Quantitative analysis reveals a mean-centered (DTW) distance of 4.2691 mm for elbow joint trajectories along the *z*-axis between the human arm and the bionic robot arm, with a deviation under 0.01. This metric significantly outperforms comparative methods, including the geometric vector method (16.0067 mm), LSTM (6.9326 mm), and genetic algorithm (5.7862 mm).

While the PSO-RF algorithm exhibits a marginally higher system latency (0.1097 s) than the geometric vector method (0.0815 s) it achieves superior balance between temporal responsiveness and spatial accuracy. The observed latency remains lower than both LSTM (0.1186 s) and genetic algorithm (0.2103 s) implementations. These results confirm that the PSO-RF algorithm effectively reconciles the precision–computational efficiency trade-off, delivering optimal comprehensive performance in motion transfer applications.

## 5. Discussion

The proposed PSO-RF hybrid algorithm demonstrates significant improvements in joint space mapping accuracy and dynamic compliance for a bionic robot arm. Experimental results indicate that the model achieves high-fidelity prediction of elbow joint angles (R^2^ = 0.932), surpassing traditional kinematic equivalence methods. The integration of PSO enables adaptive parameter tuning of the random forest model, effectively addressing error accumulation caused by marker occlusion in geometric vector analysis. Furthermore, the low operational latency (0.1097 s) ensures real-time adaptability, which is critical for human–robot collaboration tasks such as object handover or assembly.

The experiments were conducted under controlled laboratory conditions. Although a Kalman filter and an RTS smoother reduced noise and phase lag in motion capture data, the current validation focuses on predefined arm motions and so the complex multi-joint coordination motions were not fully explored, potentially limiting the generalization of the method. Additionally, the training database was established by one participant, which leads to the homogeneous physical parameters. Hence, future research should expand the motion types and the participant cohort to increase the generalization capability of the proposed PSO-RF model.

Despite differing in joint parameters, the human arm and the bionic robot arm share comparable DOF configurations, enabling them to achieve analogous motions through joint space motion transfer. The motion transfer system still exhibited persistent jitters during motion, which may be attributed to improper threshold settings in the filter implementation or the inter-joint data interference. Therefore, future research should focus on improving motion compliance through systematic parameter tuning and noise suppression strategies.

## 6. Conclusions

This paper developed a motion transfer framework for bionic robot arms by integrating a Mokov Mocap system, geometric vector-based joint angle calculation, and a hybrid PSO-optimized random forest model, which achieved high-fidelity mapping of the joint space and motion transfer from a human arm to a bionic robot arm. The PSO-RF algorithm achieved precise joint angle prediction (R^2^ = 0.932 for elbow joint angle). Then, a corresponding experimental platform was built to verify the motion transfer method proposed in this paper under various complex human arm motions. The bionic robot arm achieved similar trajectories to the human arm and exhibited a low operational latency of 0.1097 s, enabling high-precision motion transfer between the human arm and bionic robot arm. The superiority of the algorithm is verified through a comparison with existing methods. Future work will focus on further improving the motion transfer adaptability of the bionic robot arm during complex human arm motions by expanding the motion dataset and integrating impedance control. Ultimately, this method provides a scalable foundation for biomimetic robotics, with potential applications in intelligent manufacturing and medical rehabilitation.

## Figures and Tables

**Table 1:** Correspondences between motion and joint actions.

Motion	Shoulder	Elbow	Wrist
Drink water	Flexion/Extension	Flexion/Extension	Radial deviation/Ulnar deviation
	Internal/External Rotation		Dorsiflexion/Palmar flexion
Pour water	Flexion/Extension	Flexion/Extension	Radial deviation/Ulnar deviation
			Pronation/Supination
Draw a circle	Flexion/Extension	Flexion/Extension	Radial deviation/Ulnar deviation
	Adduction/Abduction		Dorsiflexion/Palmar flexion
Take the cup	Flexion/Extension	Flexion/Extension	Radial deviation/Ulnar deviation
Move the cup	Flexion/Extension	Flexion/Extension	Radial deviation/Ulnar deviation
	Adduction/Abduction		

## Data Availability

The original contributions presented in this study are included in the article. Further inquiries can be directed to the corresponding author.

## Abbreviations

The following abbreviations are used in this manuscript:

DMPs	Dynamic Movement Primitives
EMG	Electromyography
HAMPs	Human Arm Motion Patterns
Mocap	Motion Capture
RTS	Rauch–Tung–Striebel
RF	Random Forest
PSO	Particle Swarm Optimization
DTW	Dynamic Time Warping
